# Parental Dietary Knowledge, Income and Students’ Consumption of Sugar-Sweetened Beverages in China: Evidence from Longitudinal Study

**DOI:** 10.3390/nu17213356

**Published:** 2025-10-24

**Authors:** Yi Cui, Yunli Bai, Chengfang Liu

**Affiliations:** 1 China Center for Agricultural Policy, School of Advanced Agricultural Sciences, Peking University, Beijing 100871, China; cuiyicau@163.com; 2 Key Laboratory of Ecosystem Network Observation and Modeling, Institute of Geographic Sciences and Natural Resources Research, Chinese Academy of Sciences, Beijing 100101, China; ylbai.ccap@igsnrr.ac.cn; 3 United Nations Environment Programme-International Ecosystem Management Partnership (UNEP-IEMP), Beijing 100101, China

**Keywords:** sugar-sweetened beverages, parental dietary knowledge, income, longitudinal data

## Abstract

Background/Objectives: Sugar-sweetened beverage (SSB) consumption has increased globally among children and adolescents, posing significant health risks. Parental dietary knowledge and income play important roles in shaping children’s food-choice and consumption behaviors. This study aimed to examine the effects of parental dietary knowledge and income on students’ SSB consumption at both extensive and intensive margins. Methods: A two-way fixed-effects model was estimated using longitudinal data from 3962 primary and junior high school students in the Jining District of Ulanqab City, Inner Mongolia Autonomous Region, northern China, collected in 2019 and 2020. Results: SSB consumption among Chinese students increased from 2019 to 2020 in both extensive (82.51% to 86.90%) and intensive margins (686.09 mL/week to 891.21 mL/week). Each one-point increase in parental dietary knowledge score was linked to a 13.39 mL (*p* < 0.05) reduction in weekly SSB consumption, and 9.90 mL (*p* < 0.05) reduction in juice beverages, correspondingly reductions in weekly added sugar intake from SSBs (1.26 g, *p* < 0.10) and juice beverages (0.79 g, *p* < 0.05), with stronger association among rural hukou students. Parental income showed minimal association with students’ SSB consumption, but had a stronger association among rural hukou and junior high school students. Conclusions: Parental dietary knowledge plays a crucial role in reducing students’ SSB consumption, with particularly strong association in rural hukou students. Targeted interventions enhancing parental dietary knowledge could reduce SSB consumption and added sugar intake among school-aged children.

## 1. Introduction

Sugar-sweetened beverage (SSB) consumption has increased globally among children and adolescents, particularly in low- and middle-income countries [1]. Among individuals aged 3 to 19 years across 185 countries, weekly SSB consumption increased by 23%, from 2.8 servings (8 ounces per serving) in 1990 to 3.6 servings in 2018 [2], nearly twice the magnitude of increase observed among adults during the same period [3]. Children and adolescents in low- and middle-income countries consume SSBs more frequently [4]. A study across 53 low- and middle-income countries found that from 2009 to 2013, more than half (54%) of children and adolescents consumed at least one SSB daily, and 30% consumed at least twice daily [5]. China faces similar challenges: a nationally representative survey in 2013 found that nearly two-thirds of Chinese children had consumed SSBs in the past week [6]. Another study reported an upward trend in SSB consumption in China between 2004 and 2011 [7].

SSBs pose significant health risks to children and adolescents [8]. These beverages, which are high in energy density and low in nutrient value [9], have been consistently linked to adverse health outcomes across the lifespan. Evidence suggests that the consumption of SSBs is associated with an increased risk of obesity [10,11], dental caries [12,13] and behavioral problems in childhood [14,15,16]. More concerning is that early SSB consumption patterns often establishes long-term dietary patterns that increase the risk of adult obesity, type 2 diabetes, and other metabolic disorders [17,18].

Although individual [19,20,21,22,23] and environmental factors [23,24,25] influence SSB consumption, family-level determinants—particularly parents’ socioeconomic characteristics and nutrition-related knowledge—play an important role in shaping children’s dietary behavior. Parents act as both gatekeepers of their children’s food availability and role models for eating habits [26]. Little is known about how parental dietary knowledge affects children’s SSB consumption. Yet, despite extensive research on the role of parental income [7,27,28], the interaction between parental income and dietary knowledge remains underexplored, leaving uncertainty as to whether higher dietary knowledge mitigates or amplifies the effects of household economic conditions.

Building on these gaps, this study seeks to provide new empirical evidence on the relationship between parental factors and children’s SSB consumption. In particular, we address following research questions: (1) What is the association between parental dietary knowledge, income and students’ SSB consumption? (2) Does parental dietary knowledge moderate the relationship between parental income and students’ SSB consumption? (3) Does this association vary across key student subgroups? To address these questions, we applied a two-way fixed-effects (TWFE) model using a two-wave longitudinal dataset collected from northern China.

This study makes some important contributions to existing international and Chinese research. First, it moves beyond using parental education as a proxy and directly measures parental dietary knowledge to assess the association between parental dietary knowledge and students’ SSB consumption. Second, it examines the moderating role of dietary knowledge in the relationship between parental income and children’s SSB consumption, an interaction rarely tested in prior studies. Third, by applying a TWFE model to longitudinal data, this study controls for unobserved, time-invariant heterogeneity, providing more robust and reliable evidence of association than previous cross-sectional research.

## 2. Literature Review

SSB consumption among children and adolescents has been extensively studied across diverse contexts, revealing complex interactions between socioeconomic, environmental, and behavioral factors. Previous research has primarily focused on three dimensions: individual-level determinants, family-level determinants, and environmental contexts [27].

### 2.1. Indicidual Level

Studies consistently show that children’s age, gender, dietary knowledge, perceived social pressure and behavioral control ability are strong predictors of their SSB consumption [19,20,21,22,23]. Older children tend to have greater autonomy over food purchases and therefore consume more SSBs [6,7,21]. Gender differences have also been reported, with boys typically consuming higher quantities than girls [2,3]. Moreover, children’s dietary knowledge is negatively associated with SSB intake [21]. Interventions enhancing nutrition education among students have demonstrated reductions in SSB consumption [24,25].

### 2.2. Family Level

Family factors also play a crucial role in shaping children’s food choices and eating practices [26]. Extensive research has examined the effects of parents’ age, education and family income on children’s SSB consumption [7,27,28]. A close examination of family-level literature reveals at least three potential gaps. First, most existing studies emphasize the role of parental education in shaping children’s SSB consumption [2,6,7,27], while the direct influence of parental dietary knowledge remains underexplored. Unlike general educational attainment, parental dietary knowledge is more directly linked to children’s food choices, nutritional intake, and overall health outcomes [29,30]. Secondly, while some studies have examined the relationship between family income and children’s SSB consumption, little attention has been paid to how parental dietary knowledge may moderate this relationship. Some studies show positive association between family income and SSB consumption among children [7], whereas others find the opposite, reporting lower consumption among children from high-income households [31]. These mixed results may stem from the omission of parental dietary knowledge when examining the relationship between income and children’s SSB consumption. Finally, constrained by data, most studies rely on cross-sectional analyses [6,7,32,33], which help establish correlation but fail to account for unobserved time-invariant confounders, such as stable individual and family characteristics, which may bias the estimated relationships.

### 2.3. Environmental Level

Children’s food environments—both at school and in their communities—also influence SSB consumption. High densities of convenience stores, vending machines, and beverage advertisements near schools are linked to greater SSB purchases [34]. Moreover, peer and social norms strongly shape beverage choices among adolescents [35]. Some studies have shown that nutrition education targeted at children helps reduce SSB consumption [24,25].

## 3. Materials and Methods

### 3.1. Data

This study draws on a two-wave longitudinal dataset collected by the authors’ research team in 2019 and 2020 from all primary and junior high schools in Jining District, Ulanqab City, Inner Mongolia Autonomous Region, northern China. Jining District was selected as the study area for several reasons. First, it is socioeconomically representative of a typical northern Chinese district. In 2019, its per capita disposable income was 34,464 yuan, slightly higher than the national average of 30,733 yuan, reflecting a moderate economic level comparable to many urbanizing regions in northern China. Second, the dietary and nutritional characteristics of Inner Mongolia align closely with national trends, making it an ideal setting for examining SSB consumption among school-aged children. per capita added sugar consumption in Inner Mongolia was 1.3 kg in 2019, matching the national average for that year. Given that SSBs are a significant source of added sugar of children and adolescents [36], this region is particularly relevant for studying dietary patterns related to sugary beverage consumption.

In the first survey wave conducted in November 2019, we took a two-stage sampling procedure. First, we obtained a list of all 36 primary and junior high schools from the Jining District Bureau of Education and selected them as our sample schools, which included 26 primary schools and 10 junior high schools. Next, within each sample primary (junior high) school, we randomly selected two fourth-grade (seventh-grade) classes and one fifth-grade (eighth-grade) class as our sample classes, resulting in a total of 105 sample classes. It should be noted that three out of the 26 primary schools had only one fourth-grade class, which explains why the final number of sample classes was 105 rather than 108. All students present in these classes on the survey date were included, resulting in a total of 4118 sample students in 2019. All participants signed an informed consent form. The Institutional Review Board of China Agricultural University approved the protocol (Protocol ID: CAUHR-2019-005, approved on 14 October 2019).

One year later, we revisited the same 36 schools and conducted a follow-up survey of these 4118 students. As it turned out, 3962 students (96.21%) were successfully tracked in 2020, forming the final study sample for this study. A comparison between the retained and attrited samples revealed no significant differences in key study variables, including students’ SSB consumption, parental dietary knowledge and income (see Supplementary Materials Table S1). The two groups were also comparable across all measured student, parents, and school characteristics, with only three exceptions: number of siblings, parental co-residence status, and received pocket money. These limited differences suggest that sample attrition is unlikely to introduce substantial bias into our findings.

Both survey waves collected comprehensive data through structured questionnaires administered to students, their parents, head teachers, and school principals. Students, head teachers, and principals completed their questionnaires at school on the day of the survey with assistance from trained enumerators. Meanwhile, students took the parent questionnaires home to be completion and returned them to the enumerators on the following school day. The handwriting on the returned questionnaires and the enumerators’ verification confirmed that the questionnaires were self-administered by parents.

### 3.2. Variables

#### 3.2.1. Student’s SSB Consumption

Students’ SSB consumption was selected as the key dependent variable, as it directly reflects the dietary behavior of interest and is widely used in previous studies examining children’s beverage intake [4,6,31,35,37]. This study examined students’ consumption of SSBs over the past week, focusing on both extensive and intensive margins for two types of SSBs: carbonated beverages (e.g., Coke, Pepsi, or Sprite) and juice beverages (vegetable- or fruit-flavored drinks that are not 100% fruit or vegetable juice) [38]. Students were first asked whether they had consumed either type of SSB during the past week. Those who answered “yes” were then asked to retrospectively estimate the total volume consumed during that period. Based on these responses, we constructed three sets of variables, totaling nine indicators.

The first set includes three dummy variables representing the extensive margin: Dummy_total (1 if either type of SSB was consumed, 0 otherwise), Dummy_CB (1 if carbonated beverages were consumed, 0 otherwise), and Dummy_JB (1 if juice beverages were consumed, 0 otherwise). The second set measures the intensive margin based on consumption volume: ml_total (total volume of SSBs), ml_CB (volume of carbonated beverages), and ml_JB (volume of juice beverages). The third set quantifies added sugar intake. Following previous research [35], we calculated the average added sugar intake per 100 mL for each type: Sugar_total (from all SSBs), Sugar_CB (from carbonated beverages), and Sugar_JB (from juice beverages). Specifically, the proportions of added sugar were obtained from the China Food Industry Yearbook, which reports that carbonated and juice beverages contain approximately 12% and 8.5% added sugar by weight, respectively. Assuming a density of approximately 1 g/mL, the estimated added sugar content is 12 g per 100 mL for carbonated beverages and 8.5 g per 100 mL for juice beverages.

#### 3.2.2. Parental Dietary Knowledge

Parental dietary knowledge was chosen as a primary explanatory variable because parents play a crucial role in shaping children’s food choices and nutrition-related behaviors. Unlike general education, parental dietary knowledge captures more actionable aspects of health literacy that directly affect children’s consumption habits [29,30]. Parental dietary knowledge was measured based on their responses to a six-item quiz in the parent questionnaire. The quiz was adapted from the “Nutrition and Health Monitoring Survey for Students in the Rural Compulsory Education Nutrition Improvement Program,” developed by the Chinese Center for Disease Control and Prevention (see Supplementary Materials Table S2), and has been widely used in previous studies to assess dietary knowledge [35,39]. Each correct answer was awarded one point, resulting in a parental dietary knowledge (PDK) score ranging from 0 to 6. In addition, we computed the proportion of correct responses as an alternative measure of dietary knowledge.

#### 3.2.3. Parental Income

Parental income was included to represent the family’s socioeconomic status, which affects both food affordability and dietary preferences. Previous studies have shown that household income can influence children’s access to and consumption of SSBs, though the direction of the effect may vary across contexts [7,31]. Therefore, including parental income allows us to explore both its direct effect and its interaction with parental dietary knowledge. Parental income (Income) was calculated by summing the reported monthly earnings of both parents over the past month, reported in units of thousand yuan. To ensure comparability across time, parental income reported in 2020 was adjusted for inflation using the 2019 Consumer Price Index (CPI) as the base year.

#### 3.2.4. Covariates

We controlled for covariates that may influence students’ SSB consumption. Following previous studies, three individual characteristics were included: whether the student regularly received pocket money at the time of the survey [28]. the student’s dietary knowledge (measured by the same quiz as parents) [21], and whether the student lived with at least one parent (father, mother, or both).

### 3.3. Statistical Analysis

Our identification strategy utilizes the variation in parental dietary knowledge and income generated by the COVID-19 pandemic. As noted, our two-wave longitudinal data spanned the pandemic. Empirical evidence suggests that the pandemic significantly affected household income due to job losses and economic instability [40]. Simultaneously, heightened concerns about health during pandemic increased public awareness [41], prompting parents to seek health-related information [42], which may have improved their dietary knowledge. These variations present a unique opportunity to examine the effects of parental dietary knowledge and income on students’ SSB consumption.

To estimate these effects, we used a two-way fixed-effects (TWFE) model to estimate the effects of parental dietary knowledge and income on students’ SSB consumption. This model is particularly suitable for panel data, as it effectively eliminates unobserved factors that do not vary over time and mitigates potential omitted variable bias [43]. The empirical specification is as follows:

(1) $$Y_{it}=\alpha_{0}+\beta_{1}PDK_{it}+\beta_{2}Income_{it}+\gamma X_{it}+year_{t}+student_{i}+\varepsilon_{it}$$

where *i* and *t* denote student and survey wave, respectively. $Y_{it}$ denotes one of the nine SSB consumption variables constructed above for student *i* in survey wave *t*. $PDK_{it}$ denotes parental dietary knowledge score, and $Income_{it}$ represents parental income. $X_{it}$ is a vector of covariates. $year_{t}$ accounts for time fixed effects, capturing unobservable time-varying factors that may influence students’ SSB consumption. $student_{i}$ represents individual fixed effects, accounting for unobserved, time-invariant characteristics of each student. The error term $\varepsilon_{it}$ is clustered at the class level. To further explore the moderating effect of parental dietary knowledge on the relationship between parental income and students’ SSB consumption, we further include an interaction term between parental dietary knowledge and income in Equation (1).

(2) $$Y_{it}=\alpha_{0}+\beta_{1}PDK_{it}+\beta_{2}Income_{it}+\beta_{3}PDK_{it}\times Income_{it}+\gamma X_{it}+year_{t}\;+student_{i}+\varepsilon_{it}$$

Statistical significance was determined at the 5%, and 1% levels, and all analyses were performed using Stata 17.0.

## 4. Results

### 4.1. Descriptive Results

Table 1 presents summary statistics for student, parent, and school characteristics. Among the 3962 students, 47.88% were rural hukou (household registration status), 48.31% were boys, 10% were from ethnic minority groups (e.g., Mongolian, Hui, and Manchu). The average age of the students was 11.48 ± 1.55 years, with a mean of 0.58 ± 0.59 siblings. Most of students (94.52%) lived with at least one parent, and 65.55% regularly received pocket money. The average dietary knowledge score among students was 2.52 ± 1.38 (range: 0–6). Parental characteristics show that fathers had an average of 10.46 ± 3.24 years of schooling and were 42.18 ± 5.64 years old on average, while mothers had 10.01 ± 3.58 years of education and an average age of 40.03 ± 5.37 years. Among the 36 schools, 68.32% offered nutrition education courses, and 61.59% organized nutrition-related activities.

Table 2 reveals an upward trend in students’ SSB consumption between 2019 and 2020, both in extensive and intensive terms. For the extensive margin, the proportion of students consuming SSBs in the past week significantly increased from 82.51% to 86.90% (*p* < 0.01), with increases for both carbonated beverages (70.22% to 78.75%, *p* < 0.01) and juice beverages (74.10% to 81.55%, *p* < 0.01). For the intensive margin, average weekly SSB consumption in the total sample increased by 30%, from 686.09 ± 546.45 mL to 891.21 ± 620.53 mL (*p* < 0.01), approaching the global average of 892.8 mL/week across 185 countries in 2018 [2]. Specifically, carbonated beverage consumption increased by 28% (316.21 ± 317.32 mL/week to 406.18 ± 354.56 mL/week, *p* < 0.01), while juice beverage consumption increased by 31% (369.88 ± 342.67 mL/week to 485.03 ± 388.77 mL/week, *p* < 0.01). Among SSB consumers, weekly consumption grew from 831.53 ± 490.87 mL to 1025.55 ± 552.54 mL (*p* < 0.01), with similar increases observed for both carbonated (450.33 ± 288.09 mL/week to 515.81 ± 321.08 mL/week, *p* < 0.01) and juice beverages (499.13 ± 306.48 mL/week to 594.76 ± 346.50 mL/week, *p* < 0.01). Added sugar intake from SSBs also increased between 2019 and 2020. In the total sample, average added sugar intake rose from 69.39 ± 55.85 g/week to 89.67 ± 63.29 g/week (*p* < 0.01), with carbonated beverages increasing from 37.95 ± 38.08 g/week to 48.89 ± 43.54 g/week (*p* < 0.01), and juice beverages from 31.44 ± 29.13 g/week to 41.33 ± 33.67 g/week (*p* < 0.01). Among students who consumed SSBs, the average added sugar intake increased from 84.09 ± 50.43 g/week to 103.53 ± 56.62 g/week per week (*p* < 0.01), with notable increases for both carbonated beverages (54.04 ± 34.57 g to 62.09 ± 39.85 g, *p* < 0.01) and juice beverages (42.43 ± 26.06 g to 50.68 ± 30.26 g, *p* < 0.01).

Our data also reveal an increase in parental dietary knowledge but a decline in parental income during the study period. The average parental dietary knowledge score rose from 3.32 ± 1.70 points in 2019 to 3.72 ± 1.41 points in 2020 (*p* < 0.01). During the same period, the proportion of correct answers increased from 55.26% to 62.97%. However, parental monthly income fell from 6.71 ± 3.92 thousand yuan in 2019 to 6.45 ± 3.42 thousand yuan in 2020 (*p* < 0.01).

### 4.2. Regression Results of TWFE Model

Regression results from the TWFE model (1) reveal a statistically significant negative association between parental dietary knowledge and students’ SSB consumption, especially for consumption volume and added sugar intake (Table 3). Specifically, one-point increase in the parental dietary knowledge score is linked to a 13.40 mL (*p* < 0.05) decrease in total weekly SSB consumption and a 9.90 mL decrease in juice beverage consumption (*p* < 0.05). This corresponds to a 22.78 mL (approximately 3.3%) decrease in weekly SSB intake for a one-standard-deviation increase in parental dietary knowledge (1.70 points). Correspondingly, added sugar intake from juice beverages reduced by 0.79 g (*p* < 0.05) with each one-point increase in the parental dietary knowledge score. In contrast, we found no significant association between parental income and most students’ SSB consumption behavior, except for a statistically significant but negligible effect on the likelihood of students’ SSB consumption (*p* < 0.01). This suggests that in shaping children’s actual SSB consumption behaviors, knowledge held by parents is substantially more influential than their income.

Regression results from the TWFE model (2) indicate that parental dietary knowledge significantly but negligibly moderated the association between parental income and the likelihood of students consuming SSBs (Column 3 of Table 4). It suggests that the strong, negative association between parental dietary knowledge and the likelihood of students consuming SSBs holds true regardless of the family’s income level. The primary driver appears to be dietary knowledge itself, not how it interacts with economic resources. No significant moderating effects were observed for SSB consumption volume or added sugar intake.

### 4.3. Robustness Checks

We conduct two robustness checks to verify the reliability of our findings. First, we excluded 182 students (4.59%) from single-parent families to ensure the accuracy of parental income data. As shown in Table 5, the results remained consistent with the main analysis. In Model (1), parental dietary knowledge (PDK) maintained its significant negative association with students’ consumption. Each one-point increase in PDK was linked a 13.60 mL reduction in weekly SSB consumption (*p* < 0.05) and a 10.18 mL reduction in weekly juice beverages consumption (*p* < 0.05). Correspondingly, added sugar intake from juice beverages decreased by 0.81 g (*p* < 0.05) with each one-point increase in parental dietary knowledge score. Parental income remained non-significant across most outcomes. In Model (2), the interaction term between PDK and income was statistically significant for the likelihood of SSB consumption, but the effect size was negligible. No significant moderating effects were observed for SSB consumption volume or added sugar intake.

Second, we replaced parental dietary knowledge score with the proportion of correctly answered dietary questions (PDK_c) to test the robustness of the results. As shown in Table 6, the findings were broadly consistent with those from the main analysis. In Model (1), a 10-percentage-point increase in this proportion was associated with a significant 8.04 mL reduction in weekly SSB consumption (*p* < 0.05) and a 5.90 mL reduction in juice beverage consumption (*p* < 0.05). Added sugar intake also declined significantly, further confirming the negative association between parental dietary knowledge and SSB consumption. In Model (2), the interaction results suggest that for students with more knowledgeable parents, higher parental income was positively associated with the likelihood of SSB consumption (*p* < 0.01). However, the moderating relationship was not observed for SSB consumption volume or added sugar intake.

### 4.4. Heterogeneous Effects

**Figure 1:**
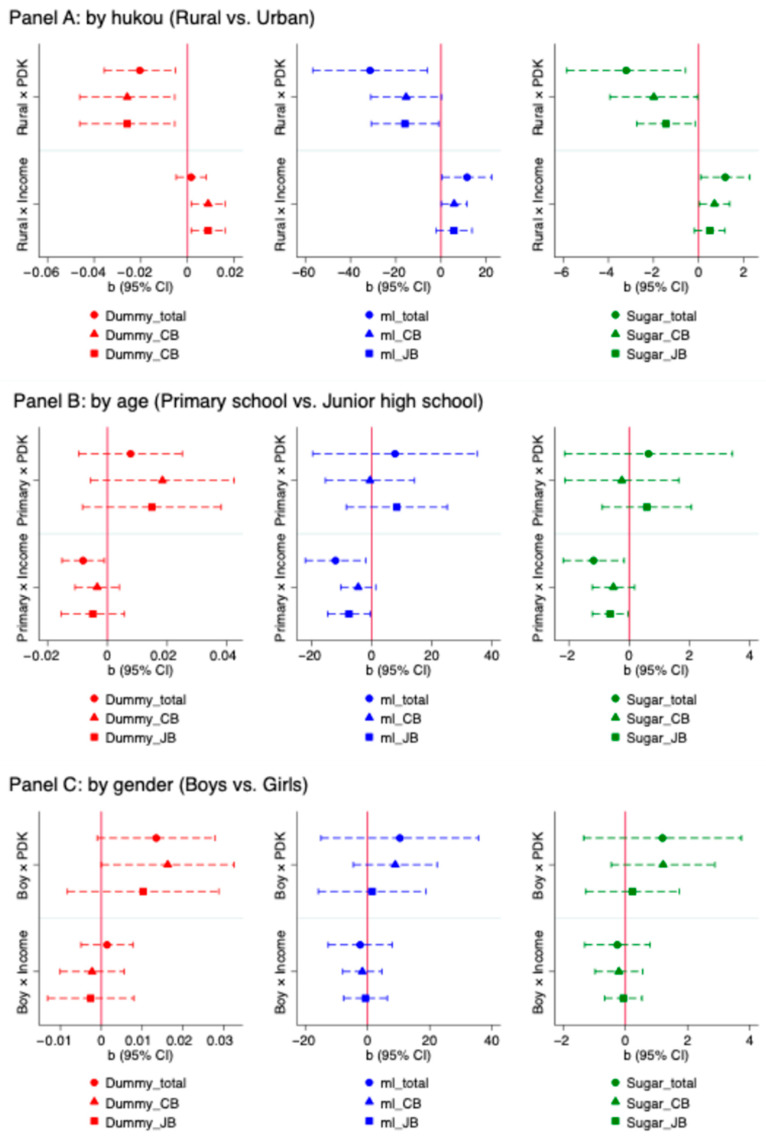
Heterogeneous association between parental income, dietary knowledge and students’ SSB consumption across hukou, age and gender groups. Notes: CI = confidence interval; PDK = parental dietary knowledge score; Income = parental income; Rural× PDK = interaction term of students’ hukou (rural) and parental dietary knowledge score; Rural × Income = interaction term of students’ hukou (rural) and parental income; Primary × PDK = interaction term of primary school and parental dietary knowledge score; Primary × Income = interaction term of primary school and parental income; Boy × PDK = interaction term of students’ gender (boy) and parental dietary knowledge score; Boy × Income = interaction term of students’ gender (boy) and parental income. The red vertical line indicates a coefficient value of zero. All models control for students’ dietary knowledge score, whether the student regularly received pocket money, whether the student lived with at least one parent, and include both individual (student) and year fixed effects. Robust standard errors are clustered at the class level. Interaction terms are estimated from the full sample (*n* = 3962) using a TWFE model. Subgroup shares: rural hukou = 48%, primary school = 63%, boys = 48%.

The effects of parental dietary knowledge and income on students’ SSB consumption may vary based on individual characteristics. For example, urban and rural hukou households exhibit distinct lifestyle patterns [44]. As children grow older, they typically gain more autonomy over pocket money and dietary choices [45]. Gender may also play a role, as girls tend to exhibit higher health awareness and stronger health-related beliefs than boys [46]. In light of these differences, we conduct heterogeneous analyses by student hukou (rural vs. urban), age (primary school vs. junior high school) and gender (boys vs. girls). Figure 1 presents the coefficients (b) and 95% confidence intervals (CIs) from a TWFE model with interaction terms for each subgroup.

Results from the heterogeneous effect analysis indicate that parental dietary knowledge and income have a stronger association with rural hukou students’ SSB consumption than their urban hukou counterparts (Panel A, Figure 1). Specifically, the negative link between parental knowledge and the likelihood of consumption was significantly stronger for rural students across all categories: total SSBs (b = −0.02; *p* < 0.01), carbonated beverages (b = −0.03; *p* < 0.01), and juice beverages (b = −0.02; *p* < 0.01). This stronger association for the rural group also extended to consumption volume and added sugar intake. For rural students, higher parental knowledge was linked to substantially larger reductions in weekly consumption of total SSBs (b = −31.38; *p* < 0.05), carbonated beverages (b = −15.44; *p* < 0.10), and juice beverages (b = −15.95; *p* < 0.05), as well as correspondingly larger decreases in added sugar intake from all three categories.

When it comes to grades, parental dietary knowledge did not show heterogeneous effects on SSB consumption between primary and junior high school students. However, the association with parental income was significantly more pronounced for junior high school students compared to their primary school peers (Panel B, Figure 1). For this older group, higher parental income was linked to a greater likelihood of consuming SSBs (b = −0.01; *p* < 0.05), higher weekly consumption of total SSBs (b = −12.03; *p* < 0.05) and juice beverages (b = −7.56; *p* < 0.05), and correspondingly higher added sugar intake from SSBs (b = −1.18; *p* < 0.05) and from juice beverages (b = −0.63; *p* < 0.05). These findings suggest that as students’ progress from primary school to junior high school, the influence of parental income on SSB consumption becomes more pronounced, likely due to increased autonomy in dietary choices and spending habits. Our analysis found no statistically significant differences in these associations by gender, though Panel C of Figure 1.

## 5. Discussion

The global increase in SSB consumption among children and adolescents poses a growing public health concern. Understanding the role of parental dietary knowledge and income in shaping children’s SSB consumption is critical for informing the design of effective interventions. Using two-wave longitudinal dataset of 3962 primary and junior high school students collected in northern China in 2019 and 2020, this study employs a two-way fixed-effects model to examine the association between parental dietary knowledge, income and students’ SSB consumption.

Our results provide evidence on both the extensive and intensive margins of SSB consumption among students. Consistent with recent findings from the United States [47,48], Australia [32] and several Central and South American countries [4], the extensive margin shows a high prevalence of SSB consumption among students. In terms of the intensive margin, our results are broadly consistent with global evidence from 185 countries [2]; however, we observe a higher consumption volume than that reported in earlier studies conducted in China [6], which found that students consumed approximately 710 mL of SSBs per week. This increase may reflect broader shifts in China’s food environment, such as the growing availability [49] and affordability of SSBs [50], as well as intensified marketing targeted at youth [51].

Meanwhile, our data reveal two contrasting trends over the study period. Parental dietary knowledge improved significantly, with the proportion of correct answers increasing from 55.26% to 62.97%. This upward trend, consistent with findings from other studies in China [52,53], likely reflects greater health awareness and more active information-seeking behaviors among parents during the COVID-19 pandemic [42]. In contrast, parental income declined during the same period, probably due to the economic disruptions and job instability caused by the pandemic [40]. These changes in dietary knowledge and income provide a valuable context for our empirical analysis, allowing us to identify their independent and interactive effects on students’ SSB consumption.

Our findings confirm that parental dietary knowledge is a crucial factor in reducing students’ SSB consumption. Evidence from other countries also supports this association between parental dietary knowledge and children’s dietary outcomes. For instance, studies in Australia [54] and Belgium [55] have shown that mothers with higher dietary knowledge are more likely to provide healthier diets for their children. While most previous research on SSB consumption has relied on general educational attainment as a proxy [2,6,7,27,56] for dietary knowledge, our analysis provides robust evidence that specific, actionable dietary knowledge serves as a more direct and meaningful predictor of children’s SSB consumption. This relationship is not only statistically significant but also practically meaningful: a one-standard-deviation increase in parental knowledge (1.7 points) is associated with a 22.8 mL weekly reduction in SSB consumption, translating to over 1.2 L annually per student. While the magnitude of this effect may seem modest, it likely represents a conservative estimate of a broader dietary improvement, as enhanced parental dietary knowledge can influence multiple unhealthy eating behaviors beyond SSB consumption [29,30]. Parents with higher dietary knowledge are more likely to create healthier food environments, establish household rules that limit children’s access to sugary and unhealthy food, and model positive dietary behaviors [28,57]. Additionally, improved dietary knowledge enables parents to better interpret food labels [58], make healthier purchasing decisions, thereby fostering sustainable improvements in children’s consumption patterns. These findings highlight the need of nutrition education programs for parents. School-based initiatives, public health campaigns, and community interventions designed to enhance parental dietary knowledge may help reduce SSB consumption and mitigate associated health risks among children and adolescents.

Our findings also indicate a statistically significant moderating effect of parental dietary knowledge on the relationship between parental income and children’s SSB consumption, although the magnitude of this effect is modest. We interpret this small effect size as a quantitative reflection of the substantial barriers parents face in translating knowledge into behavior. These barriers are twofold. Externally, it stems from an “obesogenic environment” characterized by pervasive advertising targeted at children [59] and the high accessibility of unhealthy foods [60]. Internally, it reflects the “knowledge-behavior gap”, where the cognitive load of daily life [61], stress [62], and the need for convenience [63] can override long-term health goals. Thus, the modest effect does not imply that dietary knowledge is ineffective but rather underscores the overwhelming influence of structural and contextual forces that promote unhealthy consumption. Accordingly, our findings suggest that enhancing parental dietary knowledge is a necessary but insufficient strategy; its full potential can only be realized when complemented by systemic interventions that reshape the food environment. Policies aimed at reducing environmental pressures are therefore essential, although knowledge is important, even well-informed parents need supportive environments to act on what they know.

A key finding of our study is the limited role of parental income in determining how much SSB a child consumes. While we found a statistically significant link between income and the likelihood of any SSB consumption, this association was practically negligible. More importantly, income showed no significant association with the volume of SSBs consumed or total added sugar intake. This findings contrast with earlier research conducted in Mexico [64], the United States [33] and China [7], which reported a positive association between household income and SSB consumption. We propose this reflects a modern food environment where SSBs have become both ubiquitously available and highly affordable. Due to declining real prices [50] and widespread distribution in schools and community stores [34], access to SSBs is no longer a significant barrier for lower-income families, leaving factors like parental knowledge as the more dominant drivers of children’s dietary choices. If the affordability of SSBs has effectively neutralized the influence of household income, then policies that reintroduce price as a significant factor, such as sugar tax, become a highly relevant tool [65,66]. Sugar tax would act as a market-level intervention that complements the household-level influence of parental knowledge. A dual strategy of raising the price of unhealthy options while simultaneously enhancing the knowledge of consumers could be a particularly powerful approach to public health.

This study also has several limitations. First, from a methodological standpoint, while TWFE model represents a significant advance over cross-sectional studies by controlling for time-invariant characteristics [43], it cannot account for all unobserved time-varying confounders. For instance, concurrent changes during the study period, such as evolving school wellness policies, shifts in the local food environment, or pandemic-related student stress, could also influence consumption patterns. Additionally, our study is subject to limitations regarding its scope and timing. Our focus on carbonated and juice beverages may not fully capture the rising consumption of trendy drinks like bubble tea. Furthermore, the data were collected in winter, potentially underestimating year-round consumption which may peak in summer. Finally, the findings are specific to the cultural and dietary context of northern China, which may limit their external validity. Future research should aim to address these points. Incorporating a broader range of beverages, employing longitudinal data spanning multiple seasons, and including direct measures of time-varying factors (like school policies) would strengthen the analysis. Replicating this study in diverse cultural contexts would also be essential to confirm the generalizability of our findings.

## 6. Conclusions

This study investigates the association between parental dietary knowledge, income and students’ SSB consumption in northern China using a two-wave longitudinal dataset. This study reveals an upward trend in students’ SSB consumption between 2019 and 2020, both in extensive and intensive terms. Furthermore, this study finds that parental dietary knowledge plays a crucial role in reducing students’ SSB consumption, with particularly strong effects observed in rural hukou students. These findings highlight the urgent need for targeted public health strategies to curb the rising prevalence of SSB consumption among Chinese students. Strengthening parental dietary knowledge through educational programs could effectively reduce students’ SSB consumption and added sugar intake from SSBs, particularly among rural hukou populations. Given the more pronounced impact of parental income on junior high school students, age-specific policies are necessary to address SSB consumption patterns. Combining knowledge-based strategies with policies to limit SSB access and promote healthier alternatives is essential.

## Figures and Tables

**Table 1:** Summary statistics of student, parent and school characteristics in 2019.

Variables	Mean ± SD or *n* (%)
Student characteristic	
Hukou, rural (%)	1897 (47.88)
Gender, boy (%)	1914 (48.31)
Ethnic minority, yes (%)	403 (10.20)
Age (y)	11.48 ± 1.55
Sibling (*n*)	0.58 ± 0.59
Lives with parents, yes (%)	3745 (94.52)
Pocket money, yes (%)	2597 (65.55)
Dietary knowledge score (points)	2.52 ± 1.38
Parental characteristic	
Father’s education (y)	10.46 ± 3.24
Mother’s education (y)	10.01 ± 3.58
Father’s age (y)	42.18 ± 5.64
Mother’s age (y)	40.03 ± 5.37
School characteristic	
Nutrition education, yes (%)	2707 (68.32)
Nutrition activities, yes (%)	2440 (61.59)

Note: The sample size was *n* = 3962.

**Table 2:** Summary statistics of key variables by survey wave.

Variables	Definition	Mean ± SD or *n* (%)		*p* Value
		2019	2020	
Panel A: Extensive margin (whether consumed in the past week, %)				
Dummy_total	Any SSBs, yes	3269 (82.51)	3443 (86.90)	0.00
Dummy_CB	Carbonated beverages, yes	2782 (70.22)	3120 (78.75)	0.00
Dummy_JB	Juice beverages, yes	2936 (74.10)	3231 (81.55)	0.00
Panel B: Intensive margin (consumption in the past week, milliliters)				
(1) Total sample				
ml_total	Total SSBs volume	686.09 ± 546.45	891.21 ± 620.53	0.00
ml_CB	Carbonated beverages volume	316.21 ± 317.32	406.18 ± 354.56	0.00
ml_JB	Juice beverages volume	369.88 ± 342.67	485.03 ± 388.77	0.00
(2) Conditional on consumption				
ml_total	Total SSBs volume	831.53 ± 490.87	1025.55 ± 552.54	0.00
ml_CB	Carbonated beverages volume	450.33 ± 288.09	515.81 ± 321.08	0.00
ml_JB	Juice beverages volume	499.13 ± 306.48	594.76 ± 346.50	0.00
Panel C: Intensive margin (added sugar intake, grams)				
(1) Total sample				
Sugar_total	Added sugar from SSBs	69.39 ± 55.85	89.97 ± 63.29	0.00
Sugar_CB	Added sugar from carbonated beverages	37.95 ± 38.08	48.89 ± 43.54	0.00
Sugar_JB	Added sugar from juice beverages	31.44 ± 29.13	41.33 ± 33.67	0.00
(2) Conditional on consumption				
Sugar_total	Added sugar from SSBs	84.09 ± 50.43	103.53 ± 56.62	0.00
Sugar_CB	Added sugar from carbonated beverages	54.04 ± 34.57	62.09 ± 39.85	0.00
Sugar_JB	Added sugar from juice beverages	42.43 ± 26.06	50.68 ± 30.26	0.00
Income	Parental monthly income, thousand-yuan	6.71 ± 3.92	6.45 ± 3.42	0.00
PDK	Parental dietary knowledge score (points)	3.32 ± 1.70	3.72 ± 1.41	0.00

Note. The total sample size was *n* = 3962. *p*-value was derived from *t*-tests comparing means or proportions between two waves.

**Table 3:** Two-way fixed-effects model estimates of the association between parental dietary knowledge, income and students’ SSB consumption.

Outcome Variables	PDK	Income	R^2^
	Estimate (95% CI)	Estimate (95% CI)	
Panel A: Extensive margin (consumption probability)			
Dummy_total	−0.004 (−0.01, 0.00)	0.003 ** (0.00, 0.01)	0.01
Dummy_CB	−0.007 (−0.02, 0.00)	0.000 (−0.00, 0.00)	0.03
Dummy_JB	−0.004 (−0.01, 0.01)	0.003 (−0.00, 0.01)	0.02
Panel B: Intensive margin (volume consumed)			
ml_total	−13.395 ** (−26.38, −0.41)	−0.054 (−5.45, 5.35)	0.08
ml_CB	−3.496 (−10.57, 3.58)	0.215 (−2.94, 3.37)	0.05
ml_JB	−9.899 ** (−18.23, −1.57)	−0.269 (−3.70, 3.17)	0.06
Panel C: Intensive margin (added sugar intake)			
Sugar_total	−1.261 (−2.57, 0.05)	0.003 (−0.55, 0.55)	0.08
Sugar_CB	−0.348 (−1.24, 0.54)	0.025 (−0.35, 0.40)	0.05
Sugar_JB	−0.791 ** (−1.51, −0.07)	−0.024 (−0.31, 0.27)	0.06

Note: PDK denotes the parental dietary knowledge score. Income denotes parental income. All models control for students’ dietary knowledge score, whether the student regularly received pocket money, whether the student lived with at least one parent, and include both individual (student) and year fixed effects. Robust standard errors are clustered at the class level. R^2^ denotes the within R-squared value. Sample size *n* = 3962. ** *p* < 0.05, which represent significance levels at 5%.

**Table 4:** Two-way fixed-effects model estimates of the interaction between parental dietary knowledge and income on students’ SSB consumption.

Outcome Variable	PDK	Income	PDK × Income	R^2^
	Estimate (95% CI)	Estimate (95% CI)	Estimate (95% CI)	
Panel A: Extensive margin (consumption probability)				
Dummy_total	−0.021 *** (−0.03, −0.01)	−0.004 (−0.01, 0.00)	0.002 *** (0.00, 0.00)	0.01
Dummy_CB	−0.012 (−0.03, 0.01)	−0.002 (−0.01, 0.01)	0.001 (−0.00, 0.00)	0.03
Dummy_JB	−0.016 (−0.03, 0.00)	−0.003 (−0.01, 0.01)	0.002 (−0.00, 0.00)	0.02
Panel B: Intensive margin (volume consumed)				
ml_total	−14.136 (−39.84, 11.57)	−0.398 (−11.27, 10.47)	0.113 (−3.14, 3.36)	0.08
ml_CB	−9.428 (−24.42, 5.56)	−2.539 (−9.03, 3.95)	0.903 (−1.20, 3.00)	0.05
ml_JB	−4.708 (−20.85, 11.43)	2.141 (−5.25, 9.53)	−0.790 (−2.70, 1.12)	0.06
Panel C: Intensive margin (added sugar intake)				
Sugar_total	−1.532 (−4.16, 1.10)	−0.123 (−1.23, 0.98)	0.041 (−0.30, 0.38)	0.08
Sugar_CB	−1.100 (−2.90, 0.70)	−0.325 (−1.11, 0.46)	0.115 (−0.14, 0.37)	0.05
Sugar_JB	−0.378 (−1.75, 1.00)	0.168 (−0.46, 0.79)	−0.063 (−0.23, 0.10)	0.06

Note: PDK denotes parental dietary knowledge score. Income denotes parental income. All models control for students’ dietary knowledge score, whether the student regularly received pocket money, whether the student lived with at least one parent, and include both individual (student) and year fixed effects. Robust standard errors are clustered at the class level. R^2^ denotes the within R-squared value. Sample size *n* = 3962. *** *p* < 0.01 which represent significance levels at 1% and 5%.

**Table 5:** Two-way fixed-effects model estimates of the association between parental dietary knowledge, income and their interaction on students’ SSB consumption, excluding single-parent families.

Outcome Variable	Model (1)			Model (2)	
	PDK	Income	R^2^	PDK × Income	R^2^
	Estimate (95% CI)	Estimate (95% CI)		Estimate (95% CI)	
Panel A: Extensive margin (consumption probability)					
Dummy_total	−0.004 (−0.01, 0.00)	0.004 ** (0.00, 0.01)	0.01	0.003 *** (0.00, 0.00)	0.01
Dummy_CB	−0.009 (−0.02, 0.00)	0.001 (−0.00, 0.00)	0.03	0.001 (−0.00, 0.00)	0.03
Dummy_JB	−0.004 (−0.01, 0.01)	0.003 (−0.00, 0.01)	0.02	0.002 (−0.00, 0.00)	0.02
Panel B: Intensive margin (volume consumed)					
ml_total	−13.598 ** (−26.60, −0.59)	0.776 (4.72, 6.27)	0.08	0.656 (−2.60, 3.91)	0.08
ml_CB	−3.421 (−10.53, 3.69)	0.497 (−2.68, 3.68)	0.05	1.236 (−0.90, 3.37)	0.05
ml_JB	−10.177 ** (−18.74, −1.62)	0.279 (−3.22, 3.77)	0.06	−0.580 (−2.55, 1.39)	0.06
Panel C: Intensive margin (added sugar intake)					
Sugar_total	−1.276 (−2.59, 0.04)	0.083 (−0.48, 0.64)	0.08	0.099 (−0.24, 0.44)	0.08
Sugar_CB	−0.335 (−1.24, 0.57)	0.058 (−0.32, 0.44)	0.05	0.155 (−0.10, 0.41)	0.05
Sugar_JB	−0.811 ** (−1.56, −0.07)	0.023 (−0,27, 0.32)	0.06	−0.045 (−0.21, 0.12)	0.06

Note: Model (1) reports estimate of the effects of parental dietary knowledge score (PDK) and parental income (Income) on students’ SSB consumption. Model (2) additionally includes the interaction term (PDK × Income) to examine the moderating effect of parental dietary knowledge on the income–SSB relationship. All models control for students’ dietary knowledge score, whether the student regularly received pocket money, whether the student lived with at least one parent, and include both individual (student) and year fixed effects. Robust standard errors are clustered at the class level. Robust standard errors are clustered at the class level. R^2^ denotes the within R-squared value. Sample size *n* = 3780. *** *p* < 0.01, ** *p* < 0.05, which represent significance levels at 1% and 5%.

**Table 6:** Two-way fixed-effects model estimates of the association between parental dietary knowledge (the proportion of correctly answered dietary knowledge questions), income, and their interaction on students’ SSB consumption.

Outcome Variable	Model (1)			Model (2)	
	PDK_c	Income	R^2^	PDK_c × Income	R^2^
	Estimate (95% CI)	Estimate (95% CI)		Estimate (95% CI)	
Panel A: Extensive margin (consumption probability)					
Dummy_total	−0.026 (−0.07, 0.02)	0.003 ** (0.00, 0.01)	0.01	0.015 *** (0.01, 0.02)	0.01
Dummy_CB	−0.042 (−0.11, 0.02)	0.000 (−0.00, 0.00)	0.03	0.004 (−0.00, 0.02)	0.03
Dummy_JB	−0.022 (−0.08, 0.04)	0.000 (−0.00, 0.01)	0.02	0.011 (−0.00, 0.02)	0.02
Panel B: Intensive margin (volume consumed)					
ml_total	−80.388 ** (−158.12, −2.65)	−0.047 (5.45, 5.35)	0.08	0.581 (−18.97, 20.14)	0.08
ml_CB	−21.398 (−63.71, 20.91)	0.217 (−2.93, 3.37)	0.05	5.352 (−7.27, 17.97)	0.05
ml_JB	−58.991 ** (−109.05, −8.93)	−0.264 (−3.70, 3.17)	0.06	−4.771 (−16.28, 6.74)	0.06
Panel C: Intensive margin (added sugar intake)					
Sugar_total	−7.582 (−15.44, 0.27)	0.004 (−0.55, 0.56)	0.08	0.237 (−1.81, 2.28)	0.08
Sugar_CB	−2.131 (−7.46, 3.20)	0.025 (−0.35, 0.40)	0.05	0.679 (−0.85, 2.21)	0.05
Sugar_JB	−4.705 ** (−9.05, −0.36)	−0.023 (−0.31, 0.27)	0.06	−0.379 (−1.36, 0.60)	0.06

Note: Model (1) reports estimate of the effects of the proportion of correctly answered dietary ques-tions (PDK_c) and parental income (Income) on students’ SSB consumption. Model (2) additionally includes the interaction term (PDK_c × Income) to examine the moderating effect of parental die-tary knowledge on the income–SSB relationship. All models control for students’ dietary knowledge score, whether the student regularly received pocket money, whether the student lived with at least one parent, and include both individual (student) and year fixed effects. Robust standard errors are clustered at the class level. R^2^ denotes the within R-squared value. Sample size *n* = 3962. *** *p* < 0.01, ** *p* < 0.05, which represent significance levels at 1% and 5%.

## Data Availability

Restrictions apply to dataset. The datasets presented in this article are not readily available because they are part of an ongoing study. Requests to access the datasets should be directed to Dr. Yi Cui (cuiyicau@163.com).

## Supplementary Materials

The following supporting information can be downloaded at: https://www.mdpi.com/article/10.3390/nu17213356/s1, Table S1: Comparison of SSB consumption variables between the remained and attrited samples; Table S2: The proportion of correct responses on six dietary questions in 2019 and 2020.

## Abbreviations

The following abbreviations are used in this manuscript:

SSB	Sugar-Sweetened Beverages
TWFE	Two-way fixed-effects
PDK	Parental dietary knowledge
Income	Parental income
mL	Milliliter
g	Gram
