# Supplementary material for: Parental Dietary Knowledge, Income and Students’ Consumption of Sugar-Sweetened Beverages in China: Evidence from Longitudinal Study
Source: Nutrients. 2025 Oct 24;17(21):3356. doi: 10.3390/nu17213356 (PMC12609568; doi:10.3390/nu17213356)
Supplement: Supplementary file 1 [file nutrients-17-03356-s001.zip › nutrients-3901414-supplementary.pdf]

Table S.1. Comparison of SSB consumption variables between the remained and attrited samples.

Variables	Definition	Mean ± SD or n (%)		*P*-value
		Remained n=3962	Attrited n=156	
Extensive margin (whether SSBs consumed in the past week, %)				
Dummy_total	Any SSBs, yes	3269 (82.51)	128 (82.05)	0.88
Dummy_CB	Carbonated beverages, yes	2782 (70.22)	119 (76.28)	0.10
Dummy_JB	Juice beverages, yes	2936 (74.10)	118 (75.64)	0.66
Intensive margin (SSB consumption in the past week, mL/week)				
ml_total	Total SSBs volume	686.09 ± 546.45	686.54 ± 495.59	0.99
ml_CB	Carbonated beverages volume	316.21 ± 317.32	337.66 ± 264.55	0.40
ml_JB	Juice beverages volume	369.88 ± 342.67	348.88 ± 297.85	0.45
Intensive margin (added sugar intake from SSBs, g/week)				
Sugar_total	Added sugar from SSBs	69.39 ± 55.85	70.17 ± 50.35	0.86
Sugar_CB	Added sugar from carbonated beverages	37.95 ± 38.08	40.52 ± 31.75	0.40
Sugar_JB	Added sugar from juice beverages	42.43 ± 26.05	39.20 ± 21.71	0.45
Student characteristic				
Hukou	Rural hukou, %	1621 (48.34)	68 (54.40)	0.18
Gender	Boy, %	1908 (48.22)	79 (53.38)	0.22
Ethnic minority	Yes, %	406 (10.25)	20 (12.82)	0.30
Age	Years	11.44 ± 1.56	11.61 ± 1.58	0.21
Sibling	numbers	0.80 ± 0.73	0.96 ± 0.80	0.01
Lives with parents	Yes, %	3745 (94.52)	135 (86.54)	0.00
Pocket money	Yes, %	2597 (65.55)	113 (72.44)	0.08
SDK	Student's dietary knowledge score (points)	2.52 ± 1.38	2.46 ± 1.46	0.61
Parental characteristic				
Father’s education	Years	10.40 ± 3.25	10.26 ± 3.67	0.59
Mother’s education	Years	9.87 ± 3.64	10.24 ± 3.97	0.21
Father’s age	Years	41.26 ± 5.64	41.24 ± 5.34	0.96
Mother’s age	Years	39.06 ± 5.36	39.15 ± 5.11	0.82
Income	Parental monthly income, thousand-yuan	6.71 ± 3.92	7.02 ± 3.82	0.33
PDK	Parental dietary knowledge score (points)	3.32 ± 1.70	3.23 ± 1.88	0.55
School characteristic				
Nutrition education	Yes, %	2440 (61.59)	99 (63.46)	0.57
Nutrition activities	Yes, %	2780 (68.35)	110 (70.51)	0.64

Note: *P*-values are derived from t-tests comparing means or proportions between retained and attrited samples.

Table S.2. The proportion of correct responses on six dietary questions in 2019 and 2020.

Questions	Answer options	Correct answer	Proportion of correct response	
			2019	2020
Q1: What do you think a person's health should be? (Single choice)	1= no physical illness, 2= good athletic performance, 3= Strong and physically fit, 4= not only absence of illness, but also good psychological and social adaptability, 5= other	4	72.82%	78.60%
Q2: What are the best food sources of vitamins and minerals? (Single choice)	1= beans and dairy products, 2= grains, 3= fresh vegetables and fruits, 4= meat and eggs, 5= don't know	3	53.41%	62.47%
Q3: Which of the following foods is the richest in protein? (Single choice)	1= dairy products, 2= grains, 3= vegetables and fruits, 4 = meat and eggs, 5= don't know	4	59.29%	67.54%
Q4: Which options are beneficial for growing taller? (Multiple choice)	1= drink more milk, 2= more exercise, 3= get adequate sun exposure, 4= eat more carrots, 5= don't know	1, 2, 3	27.81%	28.80%
Q5: How can iron deficiency anemia be prevented through diet? (Single choice)	1= eat more meat and fresh fruits and vegetables, 2= drink more milk, 3= eat light food, 4= don't know	1	67.34%	79.08%
Q6: What diseases are likely to be caused by frequently eating overly salty foods? (Single choice)	1= diabetes, 2= hypertension, 3= gastritis, 4= don't know	2	50.86%	61.33%

Source: The dietary knowledge questionnaire is adopted from the "Nutrition and Health Monitoring Survey for Students in the Rural Compulsory Education Nutrition Improvement Program" developed by the Chinese Center for Disease Control and Prevention.
